# Überschätzen die Lehrmittelautor:innen den authentischen Lebensweltbezug von MINT-Aufgaben? Eine Studie zur Lernendenperspektive

**DOI:** 10.1007/s40573-023-00158-9

**Published:** 2023-04-29

**Authors:** Sebastian Stuppan, Katrin Bölsterli Bardy, Andrea Maria Schmid, Markus Wilhelm

**Affiliations:** 1 Pädagogische Hochschule Luzern, Sentimatt 1, 6003 Luzern, Schweiz; 2 Pädagogische Hochschule Heidelberg, Heidelberg, Deutschland

**Keywords:** Lebensweltbezug, Lernaufgaben, Einschätzungen, Aufgabenanalysen, Perspektiven, MINT-Lernmaterialien, Relationship to daily life, Learning task, Perceptions, Task analysis, Perspectives, STEM Learning materials

## Abstract

In der Scientific Literacy und der Context-Based Science Education wird dem Lebensweltbezug eine hohe Bedeutung zugeschrieben. In vielen Forschungsprojekten zum Lebensweltbezug, ein Aspekt der Aufgabenqualität, werden schriftliche Aufgabenstellungen beurteilt und ausgewertet. Eine Schwierigkeit beim Analysieren von Aufgaben ist die Tatsache, dass die Bewertung von geschulten Beurteilenden anders ausfallen kann als durch die Zielgruppe im Unterricht. Die Dokumentenanalyse bildet das objektive Potenzial der Aufgaben aus Sicht der jeweiligen Beurteiler:innen ab. Die Einbettung der Aufgaben im Unterricht, das Niveau der Lösungen sowie die Aufgabeneinschätzung der Lernenden werden weggelassen. Studien, die sich mit der Analyse der schriftlichen Aufgaben und der Aufgabenbeurteilungen durch Lernende im Unterricht auseinandersetzen, existieren derzeit kaum. Um diese Forschungslücke zu schmälern, wird in diesem Vorhaben verglichen, wie fachdidaktische Lehrmittelautor:innen ihre entwickelten Aufgaben einschätzen und Lernende dieselben Aufgaben im Unterricht nach deren Bearbeitung wahrnehmen. Hierzu beurteilten Lernende (*N* = 805) ausgewählte MINT-Aufgaben (*N* = 16) auf deren Lebensweltbezug (min. 66, max. 182 Lernende pro Aufgabe) mithilfe des Aufgaben-Analyse-Instruments. Die Lernenden-Einschätzungen werden anschließend inferenzstatistisch der fachdidaktischen Perspektive der Lehrmittelautor:innen gegenübergestellt und diskutiert. Die Befunde dieser Studie zeigen, dass die Lernenden in den untersuchten MINT-Aufgaben die Lebenswelt divers wahrnehmen und es keine geschlechtsspezifischen Unterschiede gibt. Die Gegenüberstellung der Lernenden-Einschätzungen und der Setzung der Lehrmittelautor:innen macht deutlich, dass die Autor:innen in der Regel die Aufgaben authentischer beurteilen, als die Lernenden diese wahrnehmen. Aufgrund der doch deutlichen Diskrepanz zwischen den zwei Perspektiven wird diskutiert, welchen Mehrwert der Einbezug der Praxis in einem iterativen Verfahren beim Erstellen von Lernaufgaben haben könnte, um den Lebensweltbezug aus der Perspektive der Lernenden stringenter aufnehmen zu können.

## Abstract (en)

In scientific literacy and context-based science education, the relationship to daily life is of upmost importance. As an aspect of task quality, many research projects assess and evaluate the relationship to daily life of written tasks. A major difficulty in task analysis is that trained lecturers’ analyses can be different from those of the target group in the classroom, as document analysis shows only the objective potential of tasks from the assessors’ perspective. The embedding of tasks in lessons, the level of solutions, and task evaluation by learners have been overlooked. At present, hardly any studies exist dealing with the analysis of written tasks and task assessments by learners in the classroom. To reduce this research gap, this project compares how subject-specific education authors assess the tasks they have developed and how learners perceive the same tasks in class after they have been completed. For this purpose, learners (*N* = 805) assessed selected STEM tasks (*N* = 16) for their relationship to daily life (min. 66, max. 182 learners per task) using the Instrument to Analyse Tasks. The learners’ assessments are analyzed and discussed using inferential statistics from the perspective of teaching-material authors. This research shows that learners have diverse perceptions of the relationship to daily life in the investigated STEM tasks. Moreover, learner gender does not have a significant influence here. Comparing the learners’ and authors’ assessments of the teaching materials makes it clear that the authors generally assess the tasks to be more authentic than do the learners. Because of the obvious discrepancy between the two perspectives on tasks’ relationship to daily life, the potential benefit of iteratively including the learner’s perspective when creating tasks is discussed.

## Einleitung

Dem Lebensweltbezug wird in der Scientific Literacy und der Context-Based Science Education eine hohe Bedeutung beigemessen (Bennett et al. 2007; Bybee und McCrae 2011; Sevian et al. 2018; van Vorst et al. 2018). So ist auch bei der PISA-Studie der Lebensweltbezug als Prinzip verankert (Deutsches PISA-Konsortium und OECD 2000). Eine Verknüpfung des Unterrichts mit dem Leben außerhalb der Schule wird immer wieder gefordert und gilt als interessenförderliches Merkmal von Lernaufgaben und des Unterrichts insgesamt (Flechsig 2008; Kleinknecht et al. 2011). Blömeke et al. (2006, S. 337) und Luthiger et al. (2018) beschreiben den Lebensweltbezug als Merkmal hoher Aufgabenqualität. Dennoch gibt es Untersuchungen, die zeigen, dass eine zu alltagsnahe Lernsituation die auf Lernziele bezogene Lernleistung beeinträchtigen kann (Krajewski und Ennemoser 2010). Gemäß Büchter und Leuders (2016, S. 87) ist die Schule als didaktischer Schonraum zu verstehen und erlangt somit nie die volle Authentizität. Neubrand (2002), Maier et al. (2014) und Wilhelm et al. (2014) haben den Lebensweltbezug in ihren Kategoriensystemen in verschiedene Merkmalsausprägungen unterteilt. Dieser Unterteilung sind Stuppan, Wilhelm, Bölsterli Bardy et al. (2022) mit ihrem validierten Aufgaben-Analyse-Instrument (AAI) gefolgt. Mit dem AAI können MINT-Aufgaben bezüglich der Subskalen *Lebensweltbezug – konstruiert, Lebensweltbezug – authentisch* und *Lebensweltbezug – real *analysiert werden*.*

Eine beträchtliche Schwierigkeit beim Kategorisieren von Aufgaben ist die Unterscheidung zwischen dem objektiven Potenzial einer Aufgabe, die z. B. von Expert:innen oder Fachdidaktiker:innen eingeschätzt wird, und der tatsächlichen Realisierung der Aufgabe im Unterricht unter Berücksichtigung des Angebots-Nutzungs-Modells (Heinle et al. 2022; Maier et al. 2014, S. 11–14; Stuppan, Wilhelm, Bölsterli Bardy et al. 2022). Zahlreiche Studien rund um die Aufgabenforschung analysieren nur eine dieser Perspektiven: entweder das Potenzial der schriftlichen Aufgabe oder die Einschätzung einer Aufgabe durch Lernende oder Lehrpersonen im Unterricht. Teilweise werden Befragungen mit Lernenden durchgeführt, die ebenfalls theoretischer Natur sind. So schätzten beispielsweise in der Untersuchung von Weiss und Müller (2015) Lernende und Lehrpersonen den Lebensweltbezug von PISA-Aufgaben ein, ohne diese im Unterricht bearbeitet zu haben. Weiter beurteilen Lernende und Lehrpersonen aufgrund von forschungsökonomischen Gesichtspunkten vielfach nur wenige Aufgaben. Studien, die sich mit der Analyse der schriftlichen Aufgaben und der Aufgabenbeurteilungen durch Lernende im Unterricht auseinandersetzen, existieren derzeit kaum. Zudem wurden bisher keine Vergleiche zwischen Lernendenbeurteilungen und einer Setzung von Lehrmittelautor:innen^1^ mit dem AAI vorgenommen. Die im Folgenden vorgestellte Untersuchung setzt an den skizzierten Desideraten an. In der Studie wird der Lebensweltbezug bei 16 MINT-Aufgaben aus der Perspektive der Lernenden und der beteiligten Lehrmittelautor:innen analysiert.

## Theoretischer Hintergrund

### Lebensweltbezug im Unterricht

Seit der Gründung von Schulen als Lernstätten wird ein stärkerer Lebensweltbezug (Arbeits‑, Freizeit- und Familienwelt) mit schulischen Inhalten gefordert (Maier et al. 2010, S. 37). Weiter ist der Lebensweltbezug stark mit dem Literacy-Konzept, das die Anwendung von Wissen in möglichst realen Kontexten betont, verknüpft (Kleinknecht et al. 2011, S. 335; Schmid 2023; Sevian et al. 2018; van Vorst et al. 2015, 2018). Die Zeit der Reformpädagogik kann als Höhepunkt einer Kritik an der Lebensferne gelten. Seither wurden die Forderungen nach mehr Lebensweltbezug und Authentizität schulischen Lernens immer wieder formuliert (z. B. situiertes Lernen oder außerschulisches Lernen). Brophy und Alleman (1991, S. 19) sowie Flechsig (2008, S. 254) schlagen in ihrem Qualitätskatalog zu Lernaufgaben vor, dass Aufgaben, in Abhängigkeit vom Entwicklungsstand der Lernenden, einen Bezug zum Leben außerhalb der Schule herstellen sollen.

„Students should get to apply what they are learning to current events or other aspects of their lives outside of school (in ways that make sense, given their levels of development).“ (Brophy und Alleman 1991, S. 19)

Ferner ist der Lebensweltbezug bei den PISA-Studien (Deutsches PISA-Konsortium und OECD 2000) ein Gestaltungsprinzip von Aufgaben. Ebenso haben Blömeke et al. (2006, S. 337) den Lebensweltbezug (Repräsentation einer authentischen Situation) als didaktisches und fachliches Merkmal hoher Aufgabenqualität beschrieben. Auch Klieme et al. (2003, S. 79) und Leisen (2006, S. 6) betonen, dass Kompetenzen dann erworben und nachgewiesen werden können, wenn die Lernenden authentische Anforderungssituationen bewältigen müssen. Die Vernetzung von Wissen und Können soll nicht auf Situationen außerhalb der Schule verschoben werden. Vielmehr soll bereits beim Wissenserwerb die Vielfalt möglicher Anwendungssituationen mitgedacht werden. So sehen Herget et al. (2001) eine Aufgabe als authentisch, wenn die Lernenden diese auch tatsächlich annehmen.

Studien wie PISA (OECD 2016), ROSE (Holstermann und Bögeholz 2007; Sjøberg und Schreiner 2019) oder TIMSS (Lee und Chen 2019) und Metastudien (Krapp und Prenzel 2011; Potvin und Hasni 2014) weisen auf geschlechtsspezifische Unterschiede bezüglich fach- und themenspezifischer Interessen in naturwissenschaftlich-technischen Bereichen hin. Während Jungen sich von Themen rund um *Forschung, Technik, Maschinen, Fahrzeuge, Elektronik* sowie *gefährliche Anwendungen* begeistern lassen, wenden sich Mädchen eher davon ab und zeigen dafür in den Bereichen M*edizinische Geräte* und *Forschung verstehen* Interesse. Beide Geschlechter weisen Interesse an biologischen Themen wie *human-biologische Aspekte, Umwelt- und Gesundheitsfragen *auf. Die Berücksichtigung der unterschiedlichen thematischen Zugänge bzw. Interessen wird in der Fachdidaktik der MINT-Fächer als wesentlicher Bestandteil einer gendersensiblen Unterrichtsgestaltung betrachtet (Faulstich-Christ et al. 2010; Lembens und Bartosch 2012).

Eine Hauptanforderung beim kompetenzorientierten Unterrichten lautet: „Lernen braucht Bezug zur Lebenswelt außerhalb der Schule“ (Müller et al. 2013, S. 133). Es wird die These verfolgt, dass Unterricht auf das Lösen von realen Problemen abzielen soll, um auf das Leben vorzubereiten, und somit nützlich für den Alltag ist. Bei authentischen Problemen entstehen meist Folgefragen, beispielsweise wie das Ausgangsproblem abgewandelt, vereinfacht oder verallgemeinert werden kann. Ein authentisches Begründen oder Beweisen endet somit nicht mit der Formulierung, sondern versucht, den abgesicherten Zusammenhang tiefer zu verstehen, zu verallgemeinern und zu verknüpfen (Büchter und Leuders 2016, S. 85). Allerdings betonen Büchter und Leuders (2016, S. 87) die Grenzen der Authentizität, insofern als in der Schule organisiertes Lernen immer in einem didaktischen Schonraum stattfinde und somit niemals die volle Authentizität erlangen könne. Baumert (2002) sieht hier gleichzeitig die großen Stärken und Schwächen der Schule. So beschreiben Büchter und Leuders (2016) beispielsweise, dass „Fehler machen“ Teil eines kreativen Lernprozesses ist, der zugelassen und erwünscht ist. Krajewski und Ennemoser (2010) sowie Rey (2012) haben aufgezeigt, dass, wenn möglichst vielfältige und alltagsnahe Darstellungsmittel in einer Aufgabe vorkommen, dies wegen der Überfülle an Kontextinformationen (z. B. in Form von *seductive details*) zu einer Überlastung des Arbeitsgedächtnisses führen kann. Daher plädieren Krajewski und Ennemoser (2010, S. 213) dafür, Lernarrangements möglichst abstrakt, frei von unnötigen Informationen zu gestalten. Prediger (2009, S. 213) stellt fest: „Gerade die verständige Anwendung mathematischer Konzepte und Verfahren auf Sachsituationen scheint insbesondere schwächeren Schülerinnen und Schülern große Probleme zu bereiten.“ Um den Lebensweltbezug zu analysieren, unterscheiden deshalb Autorengruppen wie Luthiger et al. (2018), Maier et al. (2014) oder Neubrand (2002) verschiedene Aufgabenkontexte.

### Aufgabenkontext und Lebensweltbezug

Neubrand (2002, S. 112–114) unterscheidet in ihrer Analyse des Aufgabenkontextes zwischen allgemeinem und situativem Kontext. Bei einer Mathematikaufgabe kann der allgemeine Kontext einerseits ein außermathematischer Kontext oder eine innermathematische Vernetzung erfordern, während der situative Kontext aufschlüsselt, welche Art von Zusammenhang in der Aufgabe hergestellt wird. Hinsichtlich des außermathematischen Kontextes unterscheidet Neubrand (2002, S. 113–114) zwischen „real world“-Kontext, d. h., in einer Aufgabe werden realistische Daten und Zusammenhänge verwendet bzw. authentische Situationen sind dort vorgegeben, und einer Einbindung der Aufgabe in eine „scheinbare real world“. Letzteres bezeichnet Aufgaben, die mit konstruierten Daten und Zusammenhängen zum Zweck eingekleidet wurden. Weiter können Aufgaben, in denen lediglich Zahlen vorkommen, als „measurement“ gesehen werden (Neubrand 2002, S. 113). Maier et al. (2014, S. 37–38) haben sich auf die Unterscheidungen von Neubrand bezogen und definieren in ihrem Kategoriensystem den Lebensweltbezug als „Relation zwischen domänenspezifischem Fachwissen und Erfahrungs- und Lebenswelt des Jugendlichen“. Die Definitionen von Maier et al. (2014) wurden von weiteren Forschungsgruppen wie Wilhelm et al. (2014) und Luthiger et al. (2018, S. 61) aufgenommen und in die Kategoriensysteme sowie das empirisch validierte Aufgaben-Analyse-Instrument (AAI) von Stuppan, Wilhelm, Bölsterli Bardy et al. (2022) integriert. Mit dem Merkmal Lebensweltbezug wird auf die Relation zwischen dem domänenspezifischen Fachwissen (z. B. wissenschaftliche Erkenntnisse) und der individuellen Erfahrungs- und Lebenswelt der Lernenden Bezug genommen (Maier et al. 2010, S. 37–38; Stein et al. 1996, S. 486). Mit dem AAI konnten Stuppan, Wilhelm, Bölsterli Bardy et al. (2022) in ihrer Analyse von MINT-Lernaufgaben die folgenden drei Subskalen von *Lebensweltbezug *unterscheiden: *Lebensweltbezug – konstruiert, Lebensweltbezug – authentisch* und *Lebensweltbezug – real*. *Konstruiert* bedeutet, dass die dargelegte Aufgabe eine künstliche Verknüpfung zwischen dem Fachwissen und der Lebenswelt der Lernenden bildet (Maier et al. 2010, S. 38). *Authentisch* meint, dass der Kontext im Rahmen der Aufgabe Sinn ergibt (Blömeke et al. 2006; Maier et al. 2014, S. 38). Eine authentische Aufgabe könnte in Zukunft für die Lernenden relevant werden. Somit wird der Erfahrungshorizont reflektiert und in die Zukunft erweitert (Luthiger et al. 2018; Stuppan, Wilhelm und Bölsterli Bardy 2022). *Real* bedeutet, dass die Aufgabe mit den Lernenden über eine reale Fremd- oder Selbsterfahrung in Kontakt tritt (Luthiger et al. 2018, S. 61). Bei einem realen Lebensweltbezug besteht keine Differenz zwischen der Aufgabe und der Lebenswelt der Lernenden (Neubrand 2002, S. 113). Es handelt sich somit um eine Aufgabe, die tatsächlich im Leben (auch außerhalb der Schule) von den Lernenden gelöst werden muss. Wird der Lebensweltbezug von Aufgaben eingeschätzt, muss die Perspektive der Beurteilenden (wie Lernende, Lehrpersonen, Fachdidaktiker:innen oder Autor:innen von Lehrmitteln) berücksichtigt werden (Schriebl et al. 2022).

### Perspektiven der Aufgabenbeurteilung

Betz (2018, S. 226) sowie Habig et al. (2018) führen aus, dass die Wahrnehmung der Authentizität nicht nur von den Lernbedingungen, sondern auch von den Vorstellungen der Lernenden abhängig ist. So wird die Wahrnehmung einerseits durch die persönlichkeitsspezifischen Merkmale auf Lernendenebene und andererseits von den verschiedenen Merkmalen des Lernsettings bzw. der Kontextebene gesteuert (Betz et al. 2016; Habig et al. 2018; van Vorst et al. 2015). Taylor (1994) beschreibt die Problematik von Authentizität folgendermaßen:

„There is no such thing as an abstract quality ‚authenticity‘ which can be defined once and for all. Instead we should acknowledge that authenticity is a function not only of the language but also of the participants, the use to which language is put, the setting, the nature of the interaction, and the interpretation the participants bring to both the setting and the activity“. (Taylor 1994, S. 4)

Somit lässt der Lebensweltbezug die individuellen Zugänge zu fachbedeutsamen Gegenständen und Tätigkeiten offen und kann im Spektrum von „ohne Verknüpfung“ bis „tatsächlich im Leben vorkommend“ betrachtet werden. Was die einen mit ihrer Lebenswelt in Verbindung bringen können, kann für andere Lernende als Entkopplung wahrgenommen werden.

„Because learners bring different knowledge structures to a task, they may comprehend information about a certain topic differently […] what comprises a meaningful presentation for one student may not be very meaningful to another“. (Ross et al. 1986, S. 245)

In vielen Forschungsprojekten, die sich mit der Kategorisierung von Aufgaben beschäftigen, werden schriftliche Aufgabenstellungen (z. B. Aufgabenblätter, Haus- und Klassenarbeiten etc.) beurteilt. Die Dokumentenanalyse bildet jedoch nur das objektive Potenzial der Aufgaben aus Sicht der jeweiligen Beurteilenden ab (Maier et al. 2014, S. 11–12). Als Aufgabenpotenzial wird eine „in der Aufgabe angelegte, aber noch nicht realisierte Nutzungsmöglichkeit für verständnisvolle Lernprozesse“ (Hammer 2016, S. 49) verstanden. Als Beispiel seien hier die Arbeiten von Bölsterli Bardy und Wilhelm (2018), Büchter und Leuders (2016), Heinle et al. (2022), Jordan et al. (2006), Maier et al. (2010), Reinfried (2016) sowie Stuppan, Wilhelm, Bölsterli Bardy et al. (2022) genannt. Die Einbettung der Aufgabe im Unterricht sowie die Sichtweisen der Lernenden auf die Aufgabe werden nicht berücksichtigt. Als Begründung für die gewählte Methode wird u. a. angegeben, dass die Lehrpersonen den curricularen Kontext einer Aufgabe und das zu erwartende Vorwissen der Lernenden kennen (Maier et al. 2010, S. 14). Aufgrund zahlreicher Studien wie Stein et al. (1996), Neubrand (2002) und Blömeke et al. (2006) muss dies kritisch hinterfragt werden. Blömeke et al. (2006) stellen etwa ein dreistufiges Analyseverfahren vor. Zuerst wird das objektive Potenzial der Aufgaben anhand einer Dokumentenanalyse ermittelt. Anschließend wird die intendierte Aufgabenschwierigkeit durch Interviews von Lehrpersonen erhoben. Schließlich wird die Aufgabenvermittlung und -bearbeitung im Unterricht mittels Videoaufzeichnung analysiert. Dabei kam es u. a. bei einer Mathematikaufgabe, die in der Potenzialeinschätzung keine sozialen Interaktionen vorsah, zu der Situation, dass das abschließende Reflexionsgespräch sehr stark von der Lehrperson gelenkt wurde, sodass es zu einer Diskrepanz zwischen Intention und Umsetzung im Unterricht kam. Dies deutet darauf hin, dass ein Abgleich des Aufgabenpotenzials mit dem effektiven Unterricht zumindest stichprobenweise gewinnbringend sein kann. Das Vorgehen von Blömeke et al. (2006) wird einer differenzierten Aufgabenanalyse gerecht, lässt sich jedoch nur mit einem sehr hohen multimethodologischen Forschungsdesign realisieren (Maier et al. 2010, S. 14). Dennoch haben sich weitere Forschungsgruppen wie Martens et al. (2007), Nachtigall et al. (2018) oder Weiss und Müller (2015) mit einzelnen Merkmalen von Aufgaben aus Sicht von Lernenden und Lehrpersonen beschäftigt. Dabei geht es unter anderem um Aspekte der Authentizität und affektive Faktoren, wie die Motivation und Interesse. Vielfach werden in den oben genannten Untersuchungen aufgrund von forschungsökonomischen Gegebenheiten nur wenige Aufgaben oder Lernsettings analysiert. Mit den Lernmaterialien zum *„MINT unterwegs“*-Projekt werden diesbezüglich viele Optionen möglich.

### Lernmaterial – Das Projekt *„MINT unterwegs“*

*„MINT unterwegs“ *ist ein Kooperationsprojekt der Pädagogische Hochschule Luzern und der Dienststelle Volksschulbildung Luzern (DVS). Schulklassen erhalten während einer Woche Experimentierboxen und Unterrichtsmaterialien zu Bildungsstandards (EDK 2011) der Bereiche Mathematik, Informatik, Naturwissenschaften und Technik (MINT). Im Hinblick auf die allgemeine MINT-Förderung werden in Klassen des 3. bis 6. Schuljahres spezielle Projektwochen durchgeführt. Während acht Jahren (2016 bis 2023) sollen möglichst viele Schulklassen an entsprechenden Projektwochen teilnehmen und *„MINT unterwegs“* nutzen können. Während der 14 Projektwochen pro Jahr arbeiten bis zu 160 Schulklassen an einem der sechs Themen: Körper, Energie, Stoffe, Optik, Elektrizität und Robotik. Für die Lehrpersonen werden zwei Weiterbildungen durchgeführt. Die erste Weiterbildung beinhaltet konzeptionelle und fachdidaktische Hinweise, unter anderem eine Einführung in das Lernprozessmodell sowie das Erproben der *„MINT unterwegs“*-Materialien. Diese Weiterbildung findet in der Regel vier Wochen vor der Projektwoche statt. In der zweiten Weiterbildung, während der Projektwoche, finden eine Standortbestimmung und ein Ausblick statt. Die MINT-Aufgaben wurden von ausgewiesenen Fachdidaktiker:innen nach dem Lernprozessmodellansatz von Wilhelm et al. (2014) entwickelt (DVS 2020). Die Aufgabensets enthalten Konfrontationsaufgaben, Erarbeitungsaufgaben, Vertiefungsaufgaben, Übungsaufgaben, Syntheseaufgaben und teilweise Transferaufgaben (Stuppan, Wilhelm und Bölsterli Bardy 2022). Auf summative Beurteilungsaufgaben wurde größtenteils verzichtet. 2021 hat der Kanton Bern das Konzept und die Inhalte von *„MINT unterwegs“* übernommen. Zurzeit laufen Bestrebungen, die Lernmaterialien auf Französisch und Italienisch zu übersetzen.

### Das Lernprozessmodell

Um den Kompetenzerwerb über die epistemologische und didaktische Funktion von Aufgaben zu fördern, haben Wilhelm et al. (2014) und weiterführend Luthiger et al. (2018) das Luzerner Modell zur Entwicklung kompetenzfördernder Aufgabensets (LUKAS-Modell) entwickelt. Es bildet den Kompetenzerwerb als Lernprozess ab. Dies wird mit Aufgaben angestrebt, die einen kumulierenden Kompetenzaufbau ermöglichen und der Logik des Lehr-Lernprozesses entsprechen (Hattie und Yates 2015; Helmke 2015; Meyer 2016; Wellenreuther 2019). Solche Aufgabensammlungen werden von Luthiger et al. (2018, S. 33) als Aufgabensets bezeichnet. Jede einzelne Aufgabe hat somit im Unterrichtssetting eine didaktische Funktion und ist geprägt von Merkmalen, die wiederum in unterschiedlichen Ausprägungen vorliegen (Stuppan et al. under review). Das LUKAS-Modell besteht einerseits aus dem Kategoriensystem, um die Merkmale einer Aufgabe zu beschreiben, und andererseits aus dem Lernprozessmodell, das die Nutzungsseite eines Lernangebots betrachtet (Helmke 2015, S. 71) und typisiert. Im Lernprozessmodell wird der Lernprozess mit einer Konfrontationsaufgabe initiiert und mit Synthese‑/Transferaufgaben abgeschlossen (Stuppan et al. under review). Die Konfrontationsaufgabe verbindet die Lebenswelt der Lernenden mit einem Problem/Phänomen aus festgelegten Bildungsstandards. Folglich waren die Autorengruppen von *„MINT unterwegs“ *angehalten, die Konfrontationsaufgaben mit einem authentischen Lebensweltbezug zu entwickeln (DVS 2020; Wilhelm et al. 2014).

### Forschungslücke und Fragestellung

In bisherigen Untersuchungen haben geschulte Rater:innen (wie etwa Fachdidaktiker:innen oder Lehrpersonen) unter anderem das Potenzial von Lernaufgaben hinsichtlich des Lebensweltbezugs eingeschätzt (Maier et al. 2014, S. 71; Reinfried 2016; Stäudel et al. 2012; Stuppan, Wilhelm und Bölsterli Bardy 2022). Wiederum andere Forschungsgruppen haben sich mit den Perspektiven der Lernenden auseinandergesetzt und in ihren Arbeiten Unterschiede zwischen authentischen und nicht authentischen Lernorten/Medien oder Einschätzungen von Lehrpersonen ausgearbeitet (Martens et al. 2007; Nachtigall et al. 2018; Weiss und Müller 2015). Dabei wurden vielfach zwei bis drei Lernsettings in spezifischen fachdidaktischen Disziplinen untersucht (beispielsweise drei PISA-Aufgaben). Wie Lernende den Lebensweltbezug gegenüber einer Setzung von Lehrmittelautor:innen in naturwissenschaftlichen sowie technischen Aufgaben wahrnehmen, wurde bis dato ausgeklammert. Dieses Forschungsdesiderat wird in der vorliegenden Untersuchung aufgegriffen. In dieser Studie wird bei 16 MINT-Lernaufgaben der Lebensweltbezug aus der Perspektive der Lernenden sowie der Lehrmittelautor:innen analysiert. Daraus lassen sich folgende Fragen ableiten:

1. Wie beurteilen Lernende den Lebensweltbezug von MINT-Aufgaben nach deren Bearbeitung?
2. Wie unterscheiden sich die Einschätzungen betreffend Lebensweltbezug zwischen Schülerinnen und Schülern?
3. Wie unterscheiden sich die Beurteilungen der Lernenden von der Setzung der Lehrmittelautor:innen hinsichtlich des Lebensweltbezugs?

Es wird aufgrund der theoretischen Ausführungen angenommen, dass sich die Einschätzungen der Lernenden sowohl untereinander als auch von der fachdidaktischen Setzung der Lehrmittelautor:innen hinsichtlich des Lebensweltbezugs unterscheiden. Dies, weil davon ausgegangen wird, dass sich jeder Mensch selbst die Welt erschließt (Mietzel 2007; Rumpf 2010; Wilhelm und Kalcsics 2017). Mögliche genderspezifische Unterschiede in der Einschätzung des Lebensweltbezugs werden innerhalb der Schüler:innengruppen aufgrund der bisher beforschten Themeninteressen erwartet (Holstermann und Bögeholz 2007; Lee und Chen 2019; OECD 2016; Sjøberg und Schreiner 2019). Zudem sind aufgrund der Bottom-up-Perspektive der Lernenden und der Top-Down-Perspektive der Fachdidaktik Unterschiede zu erwarten (Adamina 2004; Bölsterli Bardy 2015).

## Methodik der Studie

### Instrument und Stichprobe

#### Auswahl der MINT-Aufgaben

Um die ausgeführten Forschungsfragen zu beantworten, wurden aus dem „*MINT unterwegs“*-Projekt 16 Aufgaben (Konfrontationsaufgaben) ausgewählt. Bei der Auswahl wurde darauf geachtet, dass jeweils zwei bis drei Aufgaben zu jedem Themenbereich vorliegen. Mit dieser Auswahl konnte gewährleistet werden, dass die beteiligten Lernenden nicht mehrere Fragebogen pro Tag auszufüllen hatten, und maximal an drei Befragungen in der „*MINT unterwegs“*-Projektwoche beteiligt waren. Weitere Details zu den Aufgaben sind im separaten Lernaufgabenverzeichnis aufgeführt: siehe Anhang Tab. 5.

#### Instrument und Stichprobe der Lernenden

Die Skala *Lebensweltbezug* (5-stufige Ratingskala: 0 = stimmt nicht, 1 = stimmt wenig, 2 = stimmt mittelmäßig, 3 = stimmt ziemlich, 4 = stimmt sehr) stammt aus dem Aufgaben-Analyse-Instrument (AAI) (Stuppan, Wilhelm, Bölsterli Bardy et al. 2022) und wurde für die Lernenden sprachlich marginal angepasst. Mit der Skala *Lebensweltbezug* wird auf die Relation zwischen dem domänenspezifischen Fachwissen (z. B. wissenschaftliche Erkenntnisse) und der individuellen Erfahrungs- und Lebenswelt der Lernenden Bezug genommen. Aufgrund der Annahmen aus dem AAI konnte in einem Skalen- und Fragebogen-Pretest (Datenerhebung analog wie in der Hauptstudie beschrieben, *N* = 220 Lernende aus dem „*MINT unterwegs“*-Projekt, davon 47 % Mädchen) mittels konfirmatorischer Faktorenanalyse (CFA) die Datenpassung spezifiziert und überprüft werden. Die CFA wurde mit einem robusten Maximum Likelihood (MLR) Schätzer kalkuliert. Für das Drei-Faktoren Modell in der CFA ist der χ^2^ Test signifikant (χ^2^ (24) = 43.325, *p* = 0,009). Die Fit-Indizes CFI = 0,965, SRMR = 0,043 und GFI = 0,950 weisen auf einen guten Modell-Fit für das Drei-Faktoren Modell und RMSEA = 0,066 auf einen akzeptablen Modell-Fit hin (Brown 2015; Heene et al. 2011; Hu und Bentler 1999; Marsh und Grayson 1995). Die beiden Faktoren: *Lebensweltbezug – authentisch und Lebensweltbezug – real* korrelieren signifikant (*r* = 0,79, *p* < 0,001). Aufgrund der hohen Korrelation wurde ein Zwei-Faktoren-Modell gerechnet, bei dem die Items authentisch und real auf einem gemeinsamen Faktor laden. Werden die Modelle mit einem χ^2^-Differenztest getestet, zeigt sich, dass das theoretisch angenommene dreifaktorielle Modell signifikant besser auf die Daten passt, als das Zwei-Faktoren-Modell (∆χ^2^ = 18.061, ∆*df* = 2, *p* = < 0,001). Die Subskalen weisen akzeptable Reliabilitätswerte auf (Blanz 2021): *Lebensweltbezug – konstruiert* (Cronbachs α = 0,76, Anzahl Items = 3), *Lebensweltbezug – authentisch* (α = 0,79, *n* = 3) und *Lebensweltbezug – real* (α = 0,76, *n* = 3).

Die Hauptstudie der Lernenden basiert auf einer Stichprobengröße von *N* = 805 Schülerinnen und Schülern (davon 50 % Mädchen) aus 44 Klassen. Die Lernenden besuchten das 3. bis 6. Schuljahr. Tab. 1 zeigt einen Überblick der Stichprobengröße pro Aufgabe nach Mädchen sowie Jungen. Die Schwankungen unter den einzelnen Stichproben (min. 66, max. 182 Lernende pro Aufgabe) ist dahingehend zu erklären, dass während der Untersuchung Schulschließungen als Folge der Covid-19-Pandemie stattfanden, eine projektbedingte Präferenz der Themenwahl von den Lehrpersonen selbst gegeben war und teilweise die Fragebögen der Lernenden aufgrund des Fehlens der aktiven freiwilligen Zustimmung nicht verwendet werden durften.

**Table 1:** 

Thema	Nummer und Aufgabentitel gemäß „*MINT unterwegs“*-Projekt		Stichprobengröße	
			Mädchen *n*	Jungen *n*
Körper	11	Sportarten für Sporttag wählen	64	50
	12	Was ist verletzt, was bricht	45	30
	13	Verschwundene Muskeln	53	36
Energie	21	Stromausfall	57	59
	22	Hamsterrad zur Stromerzeugung	71	63
	23	Belüftungssystem für Luca	36	30
Stoffe	31	Woraus besteht ein Wasserglace?	60	50
	32	Wer ist der Täter?	64	56
	33	Unsichtbare Stoffe	40	40
Optik	41	Hell dunkel	89	93
	42	Der tote Winkel	88	93
	43	Gegenstände, die als Lupe nutzbar sind	58	64
Elektrizität	51	Elektrisch oder nicht?	33	62
	52	Abenteuer der Familie Da Silva	36	61
Robotik	61	Rasenmähroboter im Garten	65	72
	62	Bodenroboter entdeckt die Insel	62	61

Tab. 2 zeigt eine Übersicht der drei AAI-Subskalen der Lernenden (geordnet nach *Cronbachs α)*: *Lebensweltbezug – real, Lebensweltbezug – konstruiert *und *Lebensweltbezug – authentisch*. Die Subskalen weisen akzeptable bis gute Reliabilitätswerte auf (Blanz 2021).

**Table 2:** 

Subskala	*Cronbachs α*	Items der Lernenden
Lebensweltbezug – real	0,74	Diese Aufgabe muss ich tatsächlich in meinem Leben lösen. Diese Aufgabe muss ich außerhalb der Schule auch bearbeiten. Diese Aufgabe muss ich außerhalb der Schule auch lösen.
Lebensweltbezug – konstruiert	0,80	Diese Aufgabe wurde ausgedacht. Diese Aufgabe wurde erfunden. Diese Aufgabe wurde zusammengedichtet.
Lebensweltbezug – authentisch	0,81	Diese Aufgabe könnte mit meinem echten Leben etwas zu tun haben. Diese Aufgabe könnte in meinem Alltag vorkommen. Diese Aufgabe könnte auch außerhalb meines Schulalltags vorkommen.

Anmerkung: 5‑stufige Ratingskala (von 0 = stimmt nicht bis 4 = stimmt sehr)

#### Instrument und Stichprobe der Lehrmittelautor:innen

Die Setzung der Lehrmittelautor:innen wurden mit dem von Stuppan, Wilhelm, Bölsterli Bardy et al. (2022) entwickelten und validierten AAI (*Lebensweltbezug – konstruiert*, Cronbachs α = 0,96, Anzahl Items = 3, *Lebensweltbezug – authentisch*, α = 0,93, *n* = 3 und *Lebensweltbezug – real*, α = 0,96, *n* = 3) vorgenommen. Nach einer konzeptionellen Einführung in das AAI sowie gemeinsamen Aufgabenbeurteilungen wurden die sechs Autor:innen der „*MINT unterwegs“*-Aufgaben gebeten, ihre eigenen Lernaufgaben zu beurteilen. Diese Beurteilung wird nachfolgend als Setzung der Lehrmittelautor:innen bezeichnet. Die Lehrmittelautor:innen sind mehrjährige Mitarbeiter:innen der Pädagogischen Hochschule. Sie verfügen alle über einen fachdidaktischen Hintergrund, über ein laufendes oder bereits abgeschlossenes Doktoratsstudium und eine langjährige Berufspraxis im ausgearbeiteten MINT-Themengebiet^2^.

### Datenerhebung

Die Lernenden wurden im Rahmen des Projekts *„MINT unterwegs“ *zur aktiven Studienteilnahme angefragt. Die Fragebogenerhebung (zufällige Itemreihenfolge) fand jeweils direkt nach dem Bearbeiten einer Aufgabe statt. Die Lernenden beurteilten an einem Projekttag eine Lernaufgabe bezüglich des Lebensweltbezugs; insgesamt drei Aufgaben in einer Woche, aus jeweils einem Thema. Die Teilnahme war freiwillig. Den Lehrpersonen wurde ein USB-Stick mit allen Projektunterlagen ausgehändigt. Die Lernenden mussten eine Einverständniserklärung von den Erziehungsberechtigten unterschreiben lassen (active consent). Um ein standardisiertes Vorgehen beim Ausfüllen des Fragebogens zu gewährleisten, führten alle Schulklassen zuerst einen Trainingsfragebogen aus. Die Lehrpersonen wurden in der Weiterbildung vor der *„MINT unterwegs“*-Projektwoche zum Fragebogenhandling geschult und erhielten ein schriftliches Ablaufschema. Den Lernenden wurde das Lernprozessmodell nicht erklärt. Alle erhobenen Daten wurden anonymisiert und maschinell mittels eines Auswertungsleitfadens digitalisiert. Die Setzung der Lehrmittelautor:innen wurde mit einem digital aufbereiteten Fragebogen erhoben. Sie hatten eine Schulung durchlaufen und mussten sich während des Kodierens an die Instruktionen des Testmanuals halten (Stuppan, Wilhelm, Bölsterli Bardy et al. 2022). Die Items im Fragebogen waren in einer nicht sortierten Reihenfolge.

### Statistische Auswertung

Die quantitativen Daten der Lernenden und der Lehrmittelautor:innen wurden mithilfe der Statistikprogrammumgebung R (R Development Core Team 2021) ausgewertet. Um die Daten übersichtlich vergleichen zu können, wurde dem Vorschlag von Cohen et al. (1999, S. 322–323) gefolgt und eine lineare Umrechnung der Skala Lebensweltbezug in Prozent vorgenommen (Scores Represented as the Percent of Maximum Possible, POMP). Hierzu wurde die Beurteilung der Subskalen in die Prozentanteile der Maximalpunktzahl umgerechnet.

Somit entsprechen auf der 5‑stufigen AAI-Ratingskala 0 % „stimmt nicht“, 25 % „stimmt wenig“, 50 % „stimmt mittelmäßig“, 75 % „stimmt ziemlich“ und 100 % „stimmt sehr“.

Zur Untersuchung der Forschungsfrage, ob sich bei den Lebensweltbezug-Einschätzungen ein Geschlechtsunterschied (Mädchen und Jungen) zeigt, wurde auf ein Mehrebenen-Regressionsmodell (Linear Mixed-effects Model, LMM) zurückgegriffen. Dadurch kann die Clusterung der Daten (aufgrund der Beurteilung von mehreren Aufgaben durch eine Person) berücksichtigt werden. In den drei einzelnen LMM-Analysen sind die abhängigen Variablen die Beurteilungen der Lernenden bzgl. der Subskalen: *konstruiert, authentisch* und *real* von *Lebensweltbezug. *Als unabhängige Variablen wurden die MINT-Aufgaben, das Geschlecht (Mädchen dummykodiert mit = 0 und Jungen dummykodiert mit = 1) sowie die Interaktionsterme zwischen Geschlecht und allen MINT-Aufgaben berücksichtigt. Zusätzlich wurde ein random intercept für die Lernenden geschätzt. Als Schätzung der Varianzkomponenten wurde die Restricted-Maximum-Likelihood (REML)-Methode verwendet.

Zur Analyse, inwiefern sich die Lebensweltbezug-Beurteilungen der Lernenden von den Setzungen der Lehrmittelautor:innen unterscheiden, wurden die zentralen Tendenzen geprüft und als visuelle Darstellung mit einem Boxplot-Diagramm dargestellt. Die Bedeutsamkeit der Testergebnisse wurde mit der Effektstärke nach Cohens *d* berechnet, wobei die Effektstärke gemäß Cohen (1988) folgendermaßen interpretiert werden kann: *d* = 0,20 entspricht einem kleinen Effekt, *d* = 0,50 entspricht einem mittleren Effekt; *d* = 0,80 entspricht einem starken Effekt.

## Ergebnisse

### Einschätzungen der Lernenden

Tab. 3 zeigt die Einschätzungen der Lernenden (*N* = 805) in den drei Subskalen: *konstruiert, authentisch* und *real* von *Lebensweltbezug (LWB)*. Diese Beurteilungen teilen sich nach Mädchen (M) sowie Jungen (J) auf.

**Table 3:** 

Thema	Aufg. Nr.	LWB – konstruiert		LWB – authentisch		LWB – real	
		Mädchen	Jungen	Mädchen	Jungen	Mädchen	Jungen
		*M (SD)*	*M (SD)*	*M (SD)*	*M (SD)*	*M (SD)*	*M (SD)*
Körper	11	51 (29)	48 (30)	48 (26)	48 (27)	38 (24)	41 (24)
	12	50 (28)	44 (27)	38 (26)	41 (28)	28 (25)	36 (29)
	13	48 (29)	44 (32)	43 (25)	46 (29)	34 (24)	42 (25)
Energie	21	40 (25)	44 (27)	58 (25)	59 (25)	41 (24)	44 (23)
	22	41 (27)	50 (29)	39 (28)	44 (27)	29 (24)	36 (27)
	23	43 (30)	38 (29)	38 (28)	49 (27)	27 (23)	39 (29)
Stoffe	31	45 (27)	44 (25)	49 (22)	38 (27)	41 (22)	30 (24)
	32	68 (29)	67 (32)	37 (24)	33 (26)	36 (24)	25 (24)
	33	43 (31)	54 (33)	30 (24)	40 (30)	33 (25)	28 (27)
Optik	41	46 (29)	44 (30)	47 (25)	40 (28)	41 (25)	33 (28)
	42	39 (33)	37 (34)	64 (28)	65 (32)	51 (26)	53 (33)
	43	43 (28)	49 (32)	51 (27)	39 (31)	43 (26)	34 (29)
Elektrizität	51	38 (30)	46 (31)	48 (25)	49 (23)	37 (21)	36 (25)
	52	46 (33)	52 (30)	51 (31)	49 (26)	36 (25)	37 (26)
Robotik	61	45 (28)	48 (29)	40 (25)	42 (26)	31 (20)	38 (27)
	62	60 (29)	65 (32)	29 (21)	34 (29)	27 (24)	31 (28)

Anmerkungen: *Aufg. Nr.* Aufgabennummer, *LWB* Lebensweltbezug

Gemäß LMM: Interaktion Aufgabennummer und Geschlecht in allen Fällen *p* > 0,05; die Werte sind in % als Anteil an der Maximalpunktzahl

Die Lernenden haben den *Lebensweltbezug – konstruiert* im Durchschnitt über alle untersuchten Lernaufgaben mit rund 47,5 % Zustimmung, der maximalen Punktzahl, beurteilt (*M*_M_ = 47, *SD*_M_ = 29; *M*_J_ = 48, *SD*_J_ = 30). Die höchste Zustimmung in puncto konstruierter Lebensweltbezug erhielt die Aufgabe 32 (*Stoffe – Wer ist der Täter?),* von den Mädchen mit 68 % (*SD* = 29), und die tiefste Einschätzung Aufgabe 42 (*Optik – Der tote Winkel*), von den Jungen mit 37 % (*SD* = 34).

Die Subskala *Lebensweltbezug – authentisch* erhielt von den Lernenden im Mittel 44,5 % Zustimmung (*M*_M_ = 44, *SD*_M_ = 26; *M*_J_ = 45, *SD*_J_ = 27). Als am meisten authentisch hinsichtlich Lebensweltbezug wurde Aufgabe 42 (*Optik – Der tote Winkel*) beurteilt, von den Jungen mit 65 % (*SD* = 32). Mit dem tiefsten Zustimmungswert wurde Aufgabe 62 (*Robotik – Bodenroboter entdeckt die Insel*) bedacht, von den Mädchen mit nur 29 % Zustimmung (*SD* = 21).

Der *Lebensweltbezug – real* in den untersuchten Aufgaben wurde von den Lernenden im Mittel mit 36 % (*SD*_M_ = 24; *SD*_J_ = 27) beurteilt. Dabei erhielt Aufgabe 42 (*Optik – Der tote Winkel*) am meisten Zustimmung bei der Einschätzung, ob ihr Lebensweltbezug als real wahrgenommen wird, und zwar von den Jungen mit 53 % (*SD* = 33), gegenüber Aufgabe 32 (*Stoffe – Wer ist der Täter?)*, die von den Jungen mit 25 % (*SD* = 24) als am wenigsten real in ihrem Lebensweltbezug bewertet wurde.

Um zu testen, ob die Lebensweltbezug-Einschätzungen ein Geschlechtsunterschied (Mädchen und Jungen) aufweisen, wurde auf drei LMM zurückgegriffen. Dabei erfüllen die drei Modelle folgende Voraussetzungen: Die standardisierten Residuen zeigen anhand der Histogramme sowie den Q‑Q-Plots eine annähernde Normalverteilung. Die Homoskedastizität kann aufgrund grafischer und analytischer Erkennung (mittels Breusch-Pagan-Test) als erfüllt interpretiert werden. Bei allen drei Modellen folgt anhand der Q‑Q-Plots die Verteilung der zufälligen Abweichungen von den Schätzwerten für den Achsenabschnitt und die Steigung einer angleichenden Normalverteilung. Es zeigt sich im LMM, dass die Aufgabeneinschätzungen keine signifikanten Geschlechterunterschiede in den Subskalen *Lebensweltbezug – konstruiert *(siehe Anhang Tab. 6), *Lebensweltbezug – authentisch *(siehe Anhang Tab. 7) und *Lebensweltbezug – real* (siehe Anhang Tab. 8) aufweisen.

### Einschätzung der Lernenden im Vergleich zur Setzung der Lehrmittelautor:innen

Tab. 4 zeigt im Überblick die Einschätzungen der Lernenden und die Setzung der Lehrmittelautor:innen (LMA) in den Subskalen *Lebensweltbezug – konstruiert, Lebensweltbezug – authentisch* und *Lebensweltbezug – real* bei den 16 untersuchten Aufgaben. In ihren Einschätzungen der MINT-Lernaufgaben weisen die Lernenden gegenüber den Lehrmittelautor:innen größtenteils signifikante Unterschiede bezüglich der Wahrnehmung des Lebensweltbezugs auf (Tab. 4). Keine statistisch signifikanten Unterschiede gab es bei folgenden Aufgaben und Subskalen: Aufgabe 22, 31, 33, 42 und 52 mit der Subskala *Lebensweltbezug – konstruiert*; Aufgabe 21, 41, 43 und 52 mit der Subskala *Lebensweltbezug – authentisch* sowie Aufgabe 23, 33, 51 und 52 mit der Subskala *Lebensweltbezug – real*.

Obwohl nicht bei allen Lernenden-Einschätzungen gemäß dem Shapiro-Wilk-Test eine Normalverteilung der Daten angenommen werden kann, wurden die Unterschiede in Anlehnung zu Gerl et al. (2018) sowie Sumfleth und Nakoinz (2019) mit einem Einstichproben-*t*-Test gerechnet. Eine zusätzliche Absicherung mittels nicht-parametrischer Verfahren erzielte vergleichbare Ergebnisse (auf die hier nicht weiter eingegangen wird) (Wentorf et al. 2015). Um die Alpha-Fehler-Kumulierung aufgrund wiederholter Signifikanztests auszugleichen, wurde eine Bonferroni-Korrektur angewendet (Florian et al. 2015; Watzka und Girwidz 2015). Werden die nicht signifikanten Unterschiede ausgeschlossen, zeigt sich in der Subskala *Lebensweltbezug – konstruiert*, dass die Lernenden die zu beurteilenden Aufgaben zu 55 % als stärker konstruiert wahrnehmen, als es die Setzungen der Lehrmittelautor:innen vermitteln. Bei der Subskala *Lebensweltbezug – authentisch* schätzen die Lehrmittelautor:innen ihre eigenen Aufgaben zu 92 % als mehr authentisch ein, als deren Zielgruppe es tut. Einzig Aufgabe 32 (*Stoffe – Wer ist der Täter?)*, wurde von den Lernenden als höher authentisch beurteilt. Bezüglich der Subskala *Lebensweltbezug – real* beurteilen die Lehrmittelautor:innen die Aufgaben zu 50 % signifikant höher als die Lernenden. Es zeigt sich, dass der Themenbereich „Körper“ von den Lernenden systematisch als weniger real wahrgenommen wird, als von dem Lehrmittelautor selbst eingeschätzt. Hingegen wird der Themenbereich *Robotik* von der Zielgruppe systematisch realer eingeschätzt, als es in der Setzung der Lehrmittelautorin der Fall ist.

**Table 4:** 

Thema	Aufg. Nr.	LWB – konstruiert			LWB – authentisch			LWB – real		
		Lernende	LMA-Setzung	Cohen *d*	Lernende	LMA-Setzung	Cohen *d*	Lernende	LMA-Setzung	Cohen *d*
		*M (SD)*			*M (SD)*			*M (SD)*		
Körper	11	49 (29)	17	1,13^**^	48 (26)	75	1,03^**^	39 (24)	50	0,44^**^
	12	48 (28)	25	0,82^**^	39 (27)	92	1,97^**^	31 (27)	58	1,01^**^
	13	46 (30)	8	1,26^**^	44 (27)	92	1,78^**^	37 (25)	58	0,85^**^
Energie	21	42 (26)	67	0,96^**^	58 (25)	58	0,01	43 (24)	67	1,01^**^
	22	45 (28)	42	0,13	41 (28)	58	0,61^**^	32 (26)	25	0,28^*^
	23	41 (29)	25	0,53^**^	43 (28)	92	1,77^**^	32 (26)	33	0,04
Stoffe	31	45 (26)	50	0,21	44 (25)	92	1,92^**^	36 (24)	75	1,67^**^
	32	68 (30)	92	0,80^**^	36 (25)	8	1,10^**^	31 (24)	0	1,26^**^
	33	49 (32)	50	0,04	35 (27)	75	1,47^**^	31 (26)	25	0,21
Optik	41	45 (30)	83	1,29^**^	44 (27)	42	0,07	37 (26)	25	0,46^**^
	42	38 (33)	42	0,10	64 (30)	100	1,18^**^	52 (30)	92	1,33^**^
	43	46 (30)	75	0,95^**^	45 (30)	50	0,17	38 (28)	25	0,48^**^
Elektrizität	51	43 (30)	0	1,42^**^	49 (23)	100	2,22^**^	36 (23)	33	0,12
	52	49 (31)	42	0,25	50 (28)	58	0,31	37 (25)	33	0,13
Robotik	61	47 (29)	25	0,76^**^	41 (25)	83	1,69^**^	34 (24)	25	0,39^**^
	62	63 (31)	92	0,94^**^	32 (25)	50	0,72^**^	29 (26)	0	1,11^**^

Anmerkungen: *Aufg. Nr.* Aufgabennummer, *LWB* Lebensweltbezug, *LMA* Lehrmittelautor:innen

^*^ *p* < 0,05 und ^**^ *p* < 0,001 sind durch Fettdruck hervorgehoben; die Werte sind in % als Anteil an der Maximalpunktzahl

**Figure 1:**
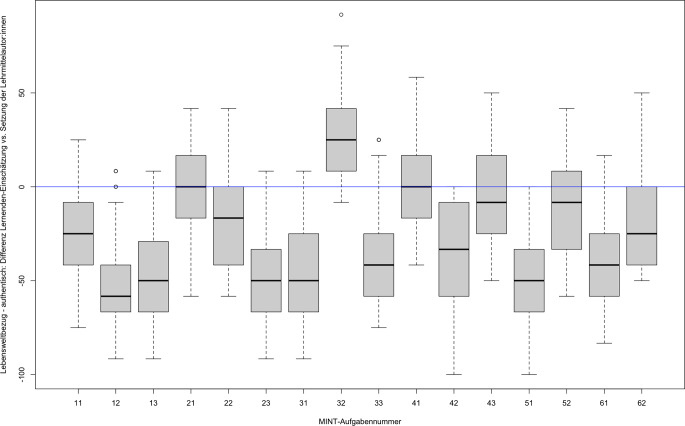

Abb. 1 verdeutlicht in Form eines Boxplot-Diagramms exemplarisch die markanten Abweichungen der Einschätzungen der Subskala *Lebensweltbezug – authentisch* der Lernenden und der Lehrmittelautor:innen (weitere Boxplot-Diagramme: siehe Anhang Abb. 2 und 3). Dabei wird die Setzung der Lehrmittelautor:innen als Nulllinie modelliert und die Differenzen der Lernenden-Beurteilungen zu den Setzungen der Lehrmittelautor:innen entsprechend als Abweichung dargestellt. Mit Ausnahme von Aufgabe 21, 41, 43 und 52 weichen die Mittelwertsunterschiede und Mediane signifikant von der Nulllinie ab. Auffallend ist, dass von den 16 untersuchten Aufgaben 11 von den Lernenden signifikant tiefer und nur eine signifikant höher beurteilt werden (vgl. Tab. 4). Es lässt sich keine Systematik hinsichtlich der MINT-Themen bzw. Aufgaben erkennen.

## Diskussion

Werden Merkmalsbereiche zur Qualität von Lernaufgaben definiert, ist der Lebensweltbezug ein zentrales Gestaltungsprinzip (Adamina und Hild 2019; Blömeke et al. 2006; Sevian et al. 2018; van Vorst et al. 2015, 2018). In bisherigen Untersuchungen zur Aufgabenqualität wurde vielfach das Potenzial von Aufgaben von geschulten Personen eingeschätzt (Heinle et al. 2022; Jordan et al. 2006; Maier et al. 2014; Stuppan, Wilhelm, Bölsterli Bardy et al. 2022). Kategorien wie der Lebensweltbezug wurden aus der Sichtweise dieser Personenkreise beurteilt. Verschiedene Autoren wie Betz (2018), Habig et al. (2018) oder Taylor (1994) haben darauf hingewiesen, dass der Lebensweltbezug individuell ist. So konnten Weiss und Müller (2015) in ihrer Untersuchung aufzeigen, dass Lehrpersonen die Authentizität von drei PISA-Aufgaben im Vergleich zur Wahrnehmung der Lernenden überschätzen. Die Beurteilungen von MINT-Aufgaben aus der Perspektive von Lernenden sowie von Lehrmittelautor:innen zu analysieren, blieb bis jetzt aus. Das Ziel der vorliegenden Studie ist es zu untersuchen, wie Lernende 16 MINT-Aufgaben im Hinblick auf den Lebensweltbezug mit dem Aufgaben-Analyse-Instrument (AAI) beurteilen, und welche Unterschiede sich in den Einschätzungen im Vergleich zu den Setzungen der Lehrmittelautor:innen ergeben. Die Ergebnisse zeigen, dass die Lernenden die 16 MINT-Aufgaben, unabhängig von ihrem angegebenen Geschlecht, als mittelmäßig authentisch wahrnehmen. Auffallend ist die hohe Variabilität in allen drei erhobenen Subskalen zum Lebensweltbezug. Werden die Einschätzungen der Lernenden den Setzungen der Lehrmittelautor:innen gegenübergestellt, wird deutlich, dass diese die Aufgaben in den meisten Fällen als höher authentisch als die Lernenden einschätzen.

### Interpretation der Ergebnisse und alternative Erklärungsversuche

Die Ergebnisse der Lernendenbefragung in den drei Subskalen von *Lebensweltbezug* zeigen hinsichtlich der Einschätzungen keine Geschlechterunterschiede. Vielmehr wird deutlich, dass, wie Kraus (2006, S. 122) ausführte, der Lebensweltbezug bereits in der Grundschule in Abhängigkeit mit den individuellen Voraussetzungen und der Umwelt steht. Somit scheint der Lebensweltbezug einer subjektiven Wahrnehmung zu unterliegen (Ross et al. 1986, S. 245). Dieses Resultat deckt sich mit aktuellen Studien zu Authentizitätseinschätzungen im Bereich von kontextualisiertem, naturwissenschaftlichem Lernen (Habig et al. 2018; van Vorst und Aydogmus 2021). Die Heterogenität der Lernenden zeigt sich auch in der Streuung der Einschätzungen innerhalb der einzelnen MINT-Aufgaben (vgl. Abb. 1). Die Varianz kann mit dem linearen gemischten Modell nach Nelson und Edwards (2015) dahingehend gestützt werden, dass die Variabilität der Lernenden-Einschätzungen bei der Subskala *Lebensweltbezug – authentisch* innerhalb der Aufgaben (σ^2^ = 0,39) größer ist, als sie zwischen den 16 Aufgaben (τ_00_ = 0,18) ist. Dabei weisen die beiden Kontrollvariablen Geschlecht und Klassenstufe in der Modellrechnung keinen signifikanten Effekt auf. Es wird vermutet, dass sich entlang der bisherigen Forschungsergebnisse zu Interessensentwicklungen genderspezifische Unterschiede bei Einbezug der Interessensmerkmalen insbesondere mit zunehmendem Alter bzw. in höheren Klassen oder unterschiedlichen zu Messzeitpunkten zeigen können (Krapp und Prenzel 2011; Lee und Chen 2019; Luo et al. 2022; OECD 2016; Potvin und Hasni 2014; Sjøberg und Schreiner 2019; van Vorst und Aydogmus 2021).

Werden die Einschätzungen der Lernenden den Setzungen der Lehrmittelautor:innen gegenübergestellt, zeigt sich, dass beide Vergleichsgruppen den *Lebensweltbezug – konstruiert *durchschnittlich mit „stimmt mittelmäßig“ beurteilen. Somit erkennen sowohl die Lernenden als auch die Lehrmittelautor:innen in den schulischen Lernaufgaben eine Form von „Einkleidung“, wie sie bereits Neubrand (2002, S. 113) in ihrem Kategoriensystem ausführte.

Wird hingegen die Subskala *Lebensweltbezug – authentisch* betrachtet, fällt auf, dass Lernende die Lernaufgaben systematisch tiefer, mit „stimmt mittelmäßig“, beurteilen als die Lehrmittelautor:innen. Bei diesen sind betreffend *Lebensweltbezug – authentisch* 9 von 16 Aufgaben mit „stimmt ziemlich“ bis „stimmt sehr“ bewertet. Möglicherweise können diese unterschiedlichen Wahrnehmungen auf ungleiche Wissensstände und Vorstellungen zurückzuführen sein (Nachtigall et al. 2018, S. 314). Ähnliche Befunde haben bereits Martens et al. (2007) sowie Gulikers et al. (2008) beschrieben. So haben Autor:innen von Aufgaben diese authentischer beurteilt als die Studierenden, die diese Aufgaben bearbeitet haben. Von vergleichbaren Resultaten berichten Weiss und Müller (2015). In den von ihnen untersuchten PISA-Aufgaben schätzten die Lernenden die Authentizität tiefer ein als die befragten Lehrpersonen. Die tiefere Beurteilung hinsichtlich Authentizität durch die Lernenden bedeutet nicht, dass die Aufgaben eine niedrige Qualität haben, sondern lediglich, dass die beiden Referenzgruppen (Lernende vs. Lehrmittelautor:innen, Fachdidaktiker:innen oder Lehrpersonen) voneinander abweichen (Weiss und Müller 2015). Dennoch müssten die Lernenden, um eine der Hauptanforderungen des kompetenzorientierten Unterrichts erfüllen zu können, den Bezug zur Lebenswelt außerhalb der Schule erkennen können (Müller et al. 2013, S. 133). Des Weiteren war die *„MINT unterwegs“*-Autorengruppe angehalten, authentische Aufgaben zu entwickeln (DVS 2020). Unter der Annahme, dass die Forderung nach authentischen MINT-Aufgaben von der Fachdidaktik selbst gestellt wird, können die Resultate der Lehrmittelautor:innen als teilweise erfüllt betrachtet werden. Wird die Wahrnehmung der Lernenden als Maßstab angelegt, müssten die Aufgaben folglich überarbeitet werden.

In einer weiterführenden Trendanalyse der Daten zeigt sich, dass sich sowohl bei den Lernenden wie auch bei den Lehrmittelautor:innen die Bewertung hinsichtlich *Lebensweltbezug – konstruiert* negativ auf die Wahrnehmung der herangezogenen Aufgaben betreffend Authentizität und Realität auswirkt. Das heißt, je stärker eine Aufgabe als konstruiert wahrgenommen wird, desto weniger authentisch oder real wird sie eingeschätzt. Weiter zeigt die Trendanalyse: Umso authentischer eine Aufgabe beurteilt wird, desto höher real wird sie wahrgenommen.

### Limitationen und Forschungsdesiderata

In der vorliegenden Studie haben die Lehrmittelautor:innen des „*MINT unterwegs*“-Projekts eine Setzung des Lebensweltbezugs bei ihren eigenen Aufgaben mithilfe des AAI vorgenommen. Dennoch wäre in zukünftigen Studien interessant zu analysieren, wie eine Gruppe von nicht involvierten Fachdidaktiker:innen den Lebensweltbezug einer Aufgabe einschätzen. Weicht ihre Beurteilung stärker von der Setzung des Lehrmittelautors bzw. der Lehrmittelautorin ab oder stärker von der Lernenden-Einschätzung? Zudem gilt es zu prüfen, wessen Ratings, die der Fachdidaktiker:innen oder jene der Lernenden, eine höhere Streuung aufweisen und wie diese Variabilitäten zu interpretieren sind. In weiterführenden Forschungsprojekten stellt sich die Frage, wie die Fachdidaktiker:innen mit der Varianz der Lernenden-Einschätzungen umgehen, da sich aufgrund der hohen Standardabweichungen empirisch keine Vereinheitlichung des Lebensweltbezugs in Aufgaben abzeichnet und dieser somit eher als abhängige, intermediäre Variable zu betrachten ist (Weiss und Müller 2015). So weisen beispielsweise die Lernenden und Lehrpersonen in der Studie von Weiss und Müller (2015) nahezu identisch hohe Standardabweichungen auf. Man war sich also innerhalb der Gruppen wie auch zwischen den Gruppen nicht einig.

Da die Lernenden beim *Lebensweltbezug – real* in den untersuchten Aufgaben größtenteils mit „stimmt wenig“ bis „stimmt mittelmäßig“ beurteilt haben, sind die Daten entsprechend asymmetrisch und rechtsschief verteilt. Dies müsste in weiteren Studien beachtet werden. Es zeigt sich jedoch, dass in vielen Studien zu Lernaufgaben kaum ein realer Lebensweltbezug gefunden wird (Gloe und Miller 2017; Heinle et al. 2022; Maier et al. 2014).

Mit der hier vorgestellten Untersuchung wurden die Lernenden quantitativ zum Lebensweltbezug befragt. Mit einer qualitativen Befragung, z. B. in Form von leitfadengestützten Interviews oder der Think-Aloud-Methode, könnten Personengruppen weitere Hintergründe zur Einschätzung der einzelnen Aufgaben sowie MINT-Themen liefern.

Weiterführende Analysen entlang des Bezugssystems für eine Beschreibung und Strukturierung von Kontextmerkmalen nach van Vorst et al. (2015) könnten differenzierte Informationen auf Schüler:innenebene, Kontextebene sowie deren Interaktion liefern. Zum Beispiel wäre vertieft zu untersuchen, inwiefern eine von den Lernenden als vielfältig und alltagsnah wahrgenommene Aufgabe sich auf die Lernleistung als auch auf kognitive und motivational-volitionale Dispositionen (wie epistemische Neugierde oder Staunen) auswirkt. Möglicherweise würde dies, wie Krajewski und Ennemoser (2010) ausführen, zu einer Überfülle an Kontextinformationen und somit zu einer Überlastung des Arbeitsgedächtnisses führen.

Da die analysierten Aufgaben Teil des Lernprozessmodells (Luthiger et al. 2018; Stuppan, Wilhelm und Bölsterli Bardy 2022) sind, wäre es interessant zu untersuchen, wie die Lernenden nach einem komplett bearbeiteten Aufgabenset die Lebenswelt der Aufgaben einschätzen. In diesem Zusammenhang wäre es aufschlussreich zu prüfen, welchen Effekt das explizite Hinweisen auf den Lebensweltbezug hat. Indem etwa der Bezug zum Leben und zum eigenen Erfahrungshorizont in die Aufgabe eingearbeitet wird, um den Lernenden bewusst zu machen, wie die Aufgabe Bezüge zum Leben schafft. In weiteren Untersuchungen wäre zu testen, inwiefern sich die Einschätzungen von Lernenden und der Fachdidaktik in Bezug auf Aspekte wie Schwierigkeit oder Verständlichkeit bei Aufgaben unterscheiden.

### Fazit

Die Ergebnisse dieser Studie legen dar, dass die Einschätzung des Lebensweltbezugs in Aufgaben nicht durch das Geschlecht der Lernenden bestimmt wird. Vielmehr unterliegt die Einschätzung wohl einer biografischen, historischen und dynamischeren intersubjektiven Wahrnehmung (Oeftering et al. 2019). Inwieweit Lehrmittelautor:innen den Lebensweltbezug ihrer eigenen Lernarrangements beurteilen können, ist aufgrund der Ergebnisse infrage zu stellen. Es zeigt sich, dass in der vorliegenden Untersuchung der Anspruch der Lehrmittelautor:innen an die Authentizität der MINT-Lernaufgaben höher ist, als von den Lernenden eingeschätzt. Um dieser Problemstellung entgegenzuwirken, wäre ein iterativer Aufgabenentwicklungsprozess zusammen mit der Praxis (u. a. Befragung von Lehrpersonen und Lernenden), wie es der Design-Based Research (DBR)-Ansatz in der Forschung verfolgt (Baumgartner et al. 2003; Reinmann 2005), in Betracht zu ziehen.

## Anhang

**Table 5:**

Thema	Nummer und Aufgabentitel		Webadresse der „*MINT unterwegs*“-Aufgabe (abgerufen am 20.09.2022)
Körper	11	01 Welche Sportarten können wir wählen?	https://mint-erleben.lu.ch/Zyklus2/show/Körper/laufen
	12	01 Der verletzte Arm	https://mint-erleben.lu.ch/Zyklus2/show/Körper/Knochenmara
	13	01 Die verschwundenen Muskeln	https://mint-erleben.lu.ch/Zyklus2/show/Körper/Gips
Energie	21	01 Luca kommt zu spät zur Schule	https://mint-erleben.lu.ch/Zyklus2/show/energie/Gesichter
	22	01 Lucas Hamster produziert Strom	https://mint-erleben.lu.ch/Zyklus2/show/energie/Energiespur
	23	01 Ein Belüftungssystem für Luca ausdenken	https://mint-erleben.lu.ch/Zyklus2/show/energie/Energiequelle
Stoffe	31	Woraus besteht eine Wasserglace?	https://mint-erleben.lu.ch/Zyklus2/show/chemie > NMG3b_Stoffe_Lehrpersonenmaterial_V40 S. 9^a^
	32	Wer ist der Täter? Teil 1: Wer hat den Drohbrief geschrieben?	https://mint-erleben.lu.ch/Zyklus2/show/chemie > NMG3b_Stoffe_Lehrpersonenmaterial_V40 S. 16^a^
	33	Sind in Wasser gelöster Zucker und Wasserdampf noch vorhanden, obwohl sie unsichtbar sind?	https://mint-erleben.lu.ch/Zyklus2/show/chemie > NMG3b_Stoffe_Lehrpersonenmaterial_V40 S. 22^a^
Optik	41	01 Hell und dunkel > Aufgabe A	https://mint-erleben.lu.ch/Zyklus2/show/Optik/lichtschatten
	42	01 Der tote Winkel	https://mint-erleben.lu.ch/Zyklus2/show/Optik/Rückspiegel
	43	01 Der vergessliche Detektiv	https://mint-erleben.lu.ch/Zyklus2/show/Optik/Lupen
Elektrizität	51	01 Was macht einen elektrischen Gegenstand aus?	https://mint-erleben.lu.ch/Zyklus2/show/Elektrizität/Elektrostatik
	52	01 Das Abenteuer der Familie Da Silva	https://mint-erleben.lu.ch/Zyklus2/show/Elektrizität/Stromkreis
Robotik	61	01 Der Rasenmähroboter im Garten	https://mint-erleben.lu.ch/Zyklus2/show/Robotik/Roboter
	62	01 Der Bodenroboter entdeckt die Schatzinsel	https://mint-erleben.lu.ch/Zyklus2/show/Robotik/Problemlösung

Anmerkung: ^a^ Aus lizenzrechtlichen Gründen ist der Zugriff nur mit einer Registrierung möglich

**Table 6:**

Prädiktoren	β	*CI*	*p*	*df*
Konstante	0,12	−0,12–0,36	0,332	1371,24
Aufg. Nr. 12	−0,09	−0,37–0,18	0,502	1097,13
Aufg. Nr. 13	−0,15	−0,41–0,11	0,257	1061,3
Aufg. Nr. 21	−0,39	−0,73–−0,05	**0,026**	1495,44
Aufg. Nr. 22	−0,34	−0,68–−0,01	**0,043**	1408,92
Aufg. Nr. 23	−0,40	−0,78–−0,03	**0,036**	1683,23
Aufg. Nr. 31	−0,23	−0,57–0,12	0,192	1444,2
Aufg. Nr. 32	0,54	0,21–0,88	**0,002**	1402,33
Aufg. Nr. 33	−0,08	−0,45–0,29	0,669	1603,87
Aufg. Nr. 41	−0,18	−0,50–0,13	0,258	1374,11
Aufg. Nr. 42	−0,38	−0,69–−0,06	**0,019**	1380,79
Aufg. Nr. 43	−0,16	−0,50–0,18	0,349	1545,05
Aufg. Nr. 51	−0,46	−0,87–−0,06	**0,026**	1477,05
Aufg. Nr. 52	−0,15	−0,54–0,25	0,465	1469,68
Aufg. Nr. 61	−0,19	−0,53–0,15	0,261	1396,32
Aufg. Nr. 62	0,28	−0,06–0,63	0,102	1416,49
Geschlecht	−0,10	−0,46–0,27	0,604	1359,45
Aufg. Nr. 12 × Geschlecht	−0,07	−0,50–0,36	0,743	1114,32
Aufg. Nr. 13 × Geschlecht	−0,05	−0,46–0,35	0,790	1092,83
Aufg. Nr. 21 × Geschlecht	0,24	−0,26–0,74	0,344	1495,02
Aufg. Nr. 22 × Geschlecht	0,40	−0,09–0,88	0,112	1435,43
Aufg. Nr. 23 × Geschlecht	−0,02	−0,58–0,54	0,952	1718,80
Aufg. Nr. 31 × Geschlecht	0,17	−0,34–0,69	0,504	1470,76
Aufg. Nr. 32 × Geschlecht	0,06	−0,44–0,56	0,826	1422,38
Aufg. Nr. 33 × Geschlecht	0,28	−0,26–0,82	0,312	1584,06
Aufg. Nr. 41 × Geschlecht	0,05	−0,41–0,51	0,820	1378,41
Aufg. Nr. 42 × Geschlecht	0,04	−0,42–0,50	0,857	1379,74
Aufg. Nr. 43 × Geschlecht	0,22	−0,27–0,71	0,372	1524,08
Aufg. Nr. 51 × Geschlecht	0,40	−0,14–0,94	0,147	1514,53
Aufg. Nr. 52 × Geschlecht	0,25	−0,29–0,78	0,363	1505,28
Aufg. Nr. 61 × Geschlecht	0,19	−0,30–0,68	0,444	1415,68
Aufg. Nr. 62 × Geschlecht	0,29	−0,20–0,79	0,246	1453,94
Zufällige Effekte				
σ^2^	0,47			
τ_00 Lernende_	0,48			
ICC	0,50			
N _Lernende_	805			
Beobachtungen	1828			
Marginal R^2^ / Konditional R^2^	0,067 / 0,537			

Anmerkungen: *LMM* Linear Mixed-effects Model, *LWB* Lebensweltbezug, *Aufg. Nr.* Aufgabennummer

**Table 7:**

Prädiktoren	β	*CI*	*p*	*df*
Konstante	0,11	−0,12–0,34	0,349	1407
Aufg. Nr. 12	−0,31	−0,59–−0,03	**0,033**	1093,69
Aufg. Nr. 13	−0,19	−0,46–0,08	0,168	1048,15
Aufg. Nr. 21	0,37	0,03–0,71	**0,033**	1559,37
Aufg. Nr. 22	−0,31	−0,63–0,01	0,058	1439,44
Aufg. Nr. 23	−0,23	−0,60–0,14	0,223	1695,77
Aufg. Nr. 31	0,02	−0,32–0,35	0,929	1472,48
Aufg. Nr. 32	−0,37	−0,70–−0,05	**0,025**	1445,82
Aufg. Nr. 33	−0,62	−0,99–−0,26	**0,001**	1652,03
Aufg. Nr. 41	−0,07	−0,38–0,23	0,646	1419,76
Aufg. Nr. 42	0,53	0,23–0,84	**0,001**	1426,24
Aufg. Nr. 43	0,05	−0,28–0,38	0,748	1588,86
Aufg. Nr. 51	0,01	−0,38–0,41	0,949	1521,24
Aufg. Nr. 52	0,11	−0,28–0,50	0,578	1515,44
Aufg. Nr. 61	−0,26	−0,59–0,06	0,112	1432,78
Aufg. Nr. 62	−0,67	−1,00–−0,34	**<** **0,001**	1457,11
Geschlecht	−0,03	−0.38–0,33	0,88	1416,66
Aufg. Nr. 12 × Geschlecht	0,08	−0,36–0,53	0,714	1136,04
Aufg. Nr. 13 × Geschlecht	0,18	−0,24–0,60	0,406	1082,08
Aufg. Nr. 21 × Geschlecht	0,01	−0,48–0,51	0,959	1553,10
Aufg. Nr. 22 × Geschlecht	0,16	−0,32–0,64	0,520	1485,24
Aufg. Nr. 23 × Geschlecht	0,27	−0,29–0,82	0,345	1727,59
Aufg. Nr. 31 × Geschlecht	−0,33	−0,83–0,16	0,189	1509,63
Aufg. Nr. 32 × Geschlecht	−0,14	−0,63–0,35	0,572	1467,54
Aufg. Nr. 33 × Geschlecht	0,31	−0,22–0,85	0,253	1636,75
Aufg. Nr. 41 × Geschlecht	−0,17	−0,62–0,28	0,461	1437,97
Aufg. Nr. 42 × Geschlecht	0,09	−0,36–0,54	0,691	1437,79
Aufg. Nr. 43 × Geschlecht	−0,34	−0,82–0,14	0,168	1575,36
Aufg. Nr. 51 × Geschlecht	0,03	−0,50–0,56	0,906	1549,71
Aufg. Nr. 52 × Geschlecht	−0,06	−0,58–0,46	0,813	1542,7
Aufg. Nr. 61 × Geschlecht	0,04	−0,44–0,51	0,870	1467,73
Aufg. Nr. 62 × Geschlecht	0,13	−0,35–0,61	0,596	1496,22
Zufällige Effekte				
σ^2^	0,50			
τ_00 Lernende_	0,40			
ICC	0,45			
N _Lernende_	805			
Beobachtungen	1805			
Marginal R^2^ / Konditional R^2^	0,105 / 0,503			

Anmerkungen: *LMM* Linear Mixed-effects Model, *Aufg. Nr.* Aufgabennummer

**Table 8:**

Prädiktoren	β	*CI*	*p*	*df*
Konstante	0,03	−0,21–0,27	0,799	1377,43
Aufg. Nr. 12	−0,29	−0,58–−0,01	**0,046**	1085,77
Aufg. Nr. 13	−0,09	−0,35–0,17	0,502	1032,21
Aufg. Nr. 21	0,19	−0,16–0,53	0,290	1509,42
Aufg. Nr. 22	−0,33	−0,67–−0,00	**0,050**	1421,70
Aufg. Nr. 23	−0,25	−0,63–0.13	0,202	1670,30
Aufg. Nr. 31	0,08	−0,26–0,43	0,630	1437,67
Aufg. Nr. 32	−0,10	−0,44–0,24	0,563	1419,51
Aufg. Nr. 33	−0,15	−0,52–0,22	0,432	1588,68
Aufg. Nr. 41	0,10	−0,21–0,42	0,515	1373,04
Aufg. Nr. 42	0,51	0,19–0,82	**0,002**	1380,49
Aufg. Nr. 43	0,16	−0,18–0,50	0,351	1546,8
Aufg. Nr. 51	−0,01	−0,42–0,40	0,967	1482,25
Aufg. Nr. 52	−0,03	−0,43–0,36	0,868	1460,17
Aufg. Nr. 61	−0,24	−0,57–0,10	0,166	1391,82
Aufg. Nr. 62	−0,41	−0,75–−0,07	**0,018**	1417,46
Geschlecht	0,11	−0,25–0,47	0,553	1375,33
Aufg. Nr. 12 × Geschlecht	0,11	−0,34–0,55	0,640	1093,88
Aufg. Nr. 13 × Geschlecht	0,16	−0,25–0,57	0,437	1071,62
Aufg. Nr. 21 × Geschlecht	−0,14	−0,64–0,36	0,592	1497,68
Aufg. Nr. 22 × Geschlecht	0,10	−0,39–0,59	0,691	1454,56
Aufg. Nr. 23 × Geschlecht	0,10	−0,46–0,66	0,714	1696,33
Aufg. Nr. 31 × Geschlecht	−0,48	−0,99–0,03	0,067	1462,87
Aufg. Nr. 32 × Geschlecht	−0,47	−0,98–0,03	0,064	1436,58
Aufg. Nr. 33 × Geschlecht	−0,45	−0,99–0,09	0,105	1573,42
Aufg. Nr. 41 × Geschlecht	−0,39	−0,85–0,07	0,097	1388,67
Aufg. Nr. 42 × Geschlecht	−0,04	−0,50–0,42	0,865	1386,74
Aufg. Nr. 43 × Geschlecht	−0,32	−0,81–0,17	0,197	1533,61
Aufg. Nr. 51 × Geschlecht	−0,15	−0,69–0,39	0,586	1513,17
Aufg. Nr. 52 × Geschlecht	−0,09	−0,62–0,45	0,753	1499,42
Aufg. Nr. 61 × Geschlecht	0,12	−0,36–0,61	0,622	1423,49
Aufg. Nr. 62 × Geschlecht	0,02	−0,47–0,52	0,933	1457,40
Zufällige Effekte				
σ^2^	0,47			
τ_00 Lernende_	0,47			
ICC	0,50			
N _Lernende_	805			
Beobachtungen	1790			
Marginal R^2^ / Konditional R^2^	0,067 / 0,532			

Anmerkungen: *LMM* Linear Mixed-effects Model, *Aufg. Nr.* Aufgabennummer

## Footnotes

1 Unter Lehrmittel wird in der Schweiz ein Lehr‑, Lern- und Arbeitsmittel verstanden, das Kompetenzen und Lerninhalte konkretisiert und für den Unterricht didaktisch aufbereitet (ilz 2022; Sandfuchs 2010). Es kann sowohl in analoger als auch in digitaler Form vorliegen.

2 Siehe Impressum auf der Website www.mint-erleben.lu.ch (abgerufen am 20. September 2022).
